# Coping with a modern pandemic-an online survey of Anesthesiologists in India during COVID-19

**DOI:** 10.1192/bjo.2021.652

**Published:** 2021-06-18

**Authors:** Punitha Chockalingam, Kalpana Balakrishnan, Priyadarshini Natarajan, Surendran Veeraiah, Revathy Rajagopal, Vinodh Kumar Elumalai

**Affiliations:** 1 Cancer Institute (WIA), Adyar; 2 South London and Maudsley NHS Foundation Trust; 3 HCG Cancer Hospital

## Abstract

**Aims:**

In 2020, India was one of the worst affected countries by COVID-19. As the pandemic spread, creating undue pressure on health care workers (HCWs), there was an urgent need for the development of appropriate interventions to protect their mental health. This study aims to study the effect of COVID-19 on the mental health of anaesthesiologists in India and factors that influence their coping behaviour.

**Method:**

The study was designed as a semi-structured, descriptive, cross-sectional, online open survey and conducted on Google forms between 21st May and 20th June 2020, among practicing anaesthesiologists across India. The participants were recruited by sending messages to their emails and through social media platforms. It created a small number of international respondents, who were also included (India = 301, rest = 23). The self-designed questionnaire had 30 questions in the form of multiple choices, checkboxes, linear scales and short comments. Informed consent was recorded at the outset. Details such as demographic characteristics, place and nature of work, pandemic related changes in duration or pattern of work, psychological symptoms during and after working hours, fears about quarantine, were collected in the survey. Statistical Analysis was performed using Statistical Package for Social Sciences (SPSS Statistics for Mac Version 21.0 IBM Corp., USA)

**Result:**

Among the 324 participating anaesthesiologists, a prevalence rate of 64.8% for stress, 51.2% for anxiety and 65.7% for depression was noted, which was double the rate from pre-pandemic studies. Those between the ages of 30 and 50 (p = 0.010 OR:2.191) and working in government run (p = 0.045 OR:2.564) COVID-19 hospitals in India (p = 0.002 OR:2.018), were particularly stressed (33.3%) and anxious (38%) than the rest. Increased workload, contracting the virus and becoming an infectious source to their family (88.6%) were their prime concern. Formulating standard operating procedures (SOP) (66.7%) and procuring personal protective equipment (PPE) (56.2%) were some of the challenges faced at work. Most of them recommended a congenial workplace (68.8%) and family support (60.8%) to help them work through their anxiety and fear, while a few reported considering leaving their career (34.8%) from fear of monetary loss and burn out (53.8%).

**Conclusion:**

COVID-19 has changed the professional and personal life of anaesthesiologists in India. Irrespective of their workplace, their fears and challenges remain universal. Early identification of anxiety and depression and providing appropriate psychological support will prevent deep and enduring damages to the lives of these professionals.

